# Correlating Volatile Lipid Oxidation Compounds with Consumer Sensory Data in Dairy Based Powders during Storage

**DOI:** 10.3390/antiox9040338

**Published:** 2020-04-20

**Authors:** Holly J. Clarke, Maurice G. O’Sullivan, Joseph P. Kerry, Kieran N. Kilcawley

**Affiliations:** 1 Food Quality and Sensory Science, Teagasc Food Research Centre, Moorepark, Fermoy, P61 C996 Co. Cork, Ireland; holly.clarke@teagasc.ie; 2 Sensory Group, School of Food and Nutritional Sciences, University College Cork, T12 R229 Cork, Ireland; maurice.osuillivan@ucc.ie; 3 Food Packaging Group, School of Food and Nutritional Sciences, University College Cork, T12 R229 Cork, Ireland; joe.kerry@ucc.ie

**Keywords:** dairy powder, infant milk formula, lipid oxidation, fatty acid, sensory, flavour, volatile profile

## Abstract

Lipid oxidation (LO) is a recognised problem in dairy powders due to the formation of volatile odour compounds that can negatively impact sensory perception. Three commercial dairy powders, fat-filled whole milk powder (FFWMP), skim milk powder (SMP), and infant milk formula (IMF), stored under different conditions (21 °C, 37 °C, or 25 °C with 50% humidity), were evaluated by consumer acceptance studies, ranked descriptive sensory analysis, and LO volatile profiling using headspace solid phase microextraction gas chromatography mass spectrometry (HS-SPME GCMS) over 16 weeks. Significant (*p* = 0.001) differences in the concentration of LO compounds and sensory perception were evident between sample types in the different storage conditions. The sensory acceptance scores for FFWMP and SMP remained stable throughout storage in all conditions, despite the increased perception of some LO products. The IMF sample was perceived negatively in each storage condition and at each time point. Overall increases in hexanal, heptanal, and pentanal correlated with “painty”, “oxidised”, “cooked”, and “caramelised” attributes in all samples. The concentration of some LO volatiles in the IMF was far in excess of those in FFWMP and SMP. High levels of LO volatiles in IMF were presumably due to the addition of polyunsaturated fatty acids (PUFA) in the formulation.

## 1. Introduction

Lipid oxidation (LO) is a well-documented cause of quality deterioration in dairy powders [1]. Oxidation of unsaturated fatty acids (FA) results in the formation of a complex mixture of primary and secondary compounds including aldehydes, ketones, and alcohols that impart off-flavours and limit shelf-life and storage stability [2]. Sensory evaluation coupled with instrumental techniques such as headspace solid-phase microextraction gas chromatography mass spectrometry (HS-SPME GCMS) have been employed for the detection and quantification of volatile aromatic compounds in various dairy products [3,4]. However, fewer studies exist that link volatile aromatic compounds to their corresponding sensory attribute(s) and/or changes in consumer perception over shelf-life [5,6]. Some studies have been carried out investigating the effects of exposing whole milk powder (WMP) to accelerated storage temperatures (45 °C) on the products’ oxidative stability [7], LO of WMP [8], and flavour and shelf-life of WMP [6]. However, less information is available on the effect of storage conditions on skim milk powder (SMP) [9,10] and infant milk formula (IMF) [11,12,13]. IMF undergoes extensive processing with the aim of simulating human breast milk from bovine milk as closely as possible [14]. However, these additional processing steps along with the addition of polyunsaturated fatty acids (PUFA) can increase LO susceptibility [15]. IMF is usually fortified with PUFA such as linoleic acid (C18:2 n6), α-linolenic (C18:3 n3), arachidonic (C20:4 n6), and docosahexaenoic acid (C22:6 n3) [16], prior to thermal processing, i.e., spray drying, for potential health benefits. Due to their unsaturated nature and low oxidative stability, PUFA are readily degraded into primary and secondary oxidation products [17], a process that can be initiated by the high inlet temperatures required for spray drying (120–180 °C) and contact with oxygen [12]. Hydroperoxides are the initial products formed by the LO cascade process and are unstable and reactive, eventually forming compounds that are known to cause off-flavours in dairy powders, such as aldehydes and ketones [17]. Thus, having more information on the volatile products of LO is important regarding the stability of products throughout their shelf-life. LO products impart specific off-flavours to milk powders; some of the most documented include “painty”, “metallic”, “fishy”, and “grassy”. Ranked descriptive analysis (RDA) has previously been used to assess consumer acceptability of dairy products [18,19]. The present study investigates the concentrations of 13 volatile LO products, in three types of dairy powders (fat-filled (FF) WMP, SMP, and IMF) by a validated HS-SPME GCMS method [20] in combination with hedonic and RDA assessment over a controlled 16 week period in different storage conditions.

## 2. Materials and Methods

### 2.1. Powder Samples

Three batches of FFWMP and SMP were obtained from local suppliers as commercial products, while three batches of IMF were purchased from local retailers. FFWMP, SMP, and IMF samples were manufactured on the following dates: December 2017, May 2018, and October 2017, respectively, each with a 24 month shelf-life. The FFWMP, SMP, and IMF were 11, 3, and 10 months into their shelf-life, respectively, at the beginning of the study. For each sample type (FFWMP, SMP, and IMF), the three batches were mixed together thoroughly to remove the batch effect and to create a bulk sample of each. The bulk sample was subsequently separated into four 1.5 kg lots and placed in light-omitting sealed bags at the beginning of the study (T0). One bag of each sample was stored in one of the four storage treatments; –18 °C (control), 21 °C (ambient), 37 °C (accelerated), and 25 °C with humidity controlled at 50% (humidity) resulting in 12 samples (3 × 4) in total. Samples were stored at –18 °C (freezer) and 21 °C and 37 °C (incubation rooms) at the Teagasc Food Research Centre (Fermoy, Co. Cork, Ireland), and samples kept at 25 °C with humidity controlled at 50% were stored in a Binder KBF P Series Humidity Test Chamber (Binder GmbH, Tuttlingen, Germany) located at University College Cork (Cork, Ireland). Ambient is denoted as AM, control as CON, accelerated as ACC, and humidity as HUM throughout the study, unless otherwise stated. Volatile data were undertaken at T0, T2, T4, T6, T8, T10, T12, T14, and T16, which represented the number of weeks of storage. Sensory analysis was carried out at T4, T8, T12, and T16 for practical purposes, and sensory and volatile data were correlated at T4, T8, T12, and T16. An additional six IMF samples were purchased from local retail units and immediately analysed for LO volatiles (in triplicate) for comparative purposes, as it was necessary to get a better understanding of the potential LO range in these products as little or no data were available.

### 2.2. Compound Selection

Thirteen volatile aromatic compounds including seven aldehydes, hexanal, pentanal, heptanal, octanal, (E)-2-nonenal, 2,4-decadienal, and undecanal, four ketones, 2-heptanone, 2-nonanone, 2-pentanone, and 3-octen-2-one, and two alcohols, 1-heptanol and 1-pentanol, known to be important for the sensory perception of dairy products, were selected for quantification based on current literature [17,18,21]. Authentic standards for each of the target compounds and internal standard compounds (isovaleraldehyde, 2-methyl-3-heptanone, and 4-methyl-2-pentanol) were purchased from Merck (Arklow, Wicklow, Ireland).

### 2.3. Powder Composition

Each sample was analysed for fat, protein, lactose, true protein, and casein content using a Bentley DairySpec FT (Technopath Distribution, Ballina, Co. Tipperary, Ireland). Samples were reconstituted to 10% total solids for SMP and 13% for FFWMP and IMF, as per the manufacturer’s instructions using distilled water (dH_2_O) 24 h prior to analysis. Samples were heated to ~40 °C immediately before sampling. Results were expressed as the averages of 2 replicates.

### 2.4. Microbial Analysis

The pour plate method was used to estimate the total bacterial count of the reconstituted milk powder samples prior to each sensory evaluation session. Serial dilutions from 10^0^–10^4^ were prepared in 9 mL maximum recover diluent (Thermo Fisher Scientific Oxoid CM0733, Basingstoke, UK). One millilitre of each dilution was pipetted onto sterile petri dishes and covered with warm (45 ± 2 °C), sterile milk plate count agar (15 mL) (MPCA; Thermo Fisher Scientific Oxoid CM0681, Basingstoke, UK). The mixture was allowed to cool and solidify, and plates were incubated for 72 h at 30 °C. Analysis was performed in duplicate.

### 2.5. Milk Powder Colour Measurements

Colour measurements were performed on each of the 12 milk powder samples according to the CIE Lab system (CIE, 1978; L* is a measure of lightness; a* is a measure of green-to-red colour on a negative to positive scale, respectively; and b* is a measure of blue-to-yellow colour on a negative to positive scale, respectively), using a Minolta colorimeter (Minolta Camera, Osaka, Japan). Samples were reconstituted in dH_2_O 24 h prior to analysis and chilled at 4 °C. Approximately 2 mL of sample were placed in a spectrophotometric cuvette 1 h prior to analysis. Results were expressed as the average of triplicate measurements of each liquid sample [18].

### 2.6. Fatty Acid Analysis

Lipid extraction and methyl ester derivatisation of triglycerides were carried out as per De Jong and Badings [22] and O‘Callaghan et al. [23]. All milk powder samples were reconstituted to 12% total solids 1 h prior to analysis, and 10 mL of reconstituted milk powder were used for analysis. Chromatographic conditions were also as outlined by O‘Callaghan et al. [23]. Briefly, analysis was performed on an Agilent 7890A gas chromatograph, equipped with an Agilent 7693 autosampler (Agilent Technologies Ltd., Cork, Ireland) and flame ionised detector (FID). The column was a Select FAME capillary column (100 m × 250 µm internal diameter (I.D.), 0.25 µm phase thickness, part number: CP7420) (Agilent Technologies Ltd., Cork, Ireland). The injector was held at 250 °C for the entire run and was operated in split mode using a split ratio of 1:10. The inlet liner was a split gooseneck liner (part no.: 8004–0164, Agilent Technologies Ltd., Cork, Ireland). The column oven was held at 80 °C for 8 min, raised to 200 °C at 8.5 °C/min, and held for 55 min. The total runtime was 77.12 min. The FID was operated at 300 °C. The carrier gas was hydrogen and was held at a constant flow of 1.0 mL/min. Results were processed using OpenLab CDS Chemstation edition software Version Rev.C.01.04 (35) (Agilent Technologies Ltd., Cork, Ireland).

A 37 component fatty acid methyl ester (FAME) reference mix containing C4:0 to C24:0 (Part Number 35077) (Thames Restek Ltd., Buckinghamshire, UK) was analysed as an in-run quality control sample, with the FAME present at a 60–180 ppm concentration. This was used to ensure accurate quantitation was achieved throughout sample analysis. The FAME mix was analysed once every five samples in the sequence. Accuracy was monitored by comparing the measured concentration of the FAME mix against its true concentration.

### 2.7. Volatile Analysis

#### 2.7.1. HS-SPME Conditions

Headspace solid-phase microextraction (HS-SPME) analysis was carried out using an HS-SPME method optimised for the detection and quantification of LO compounds in WMP as per Clarke et al. [20]. Briefly, 2.4 g of each powder sample were weighed out directly into La-Pha-Pack headspace vials (20 mL) with magnetic screw caps and silicone/polytetrafluoroethylene 1.3 mm 45° Shore A septa (Apex Scientific Ltd., Maynooth Co., Kildare, Ireland). To each sample, 250 μL of the internal standard mixture (2-methyl-3-heptanone, 4-methyl-2-pentanol, and isovaleraldehyde) prepared at 0.001% (*w/v*) in dH_2_O were added along with 3.5 mL dH_2_O. A calibration curve was prepared by spiking a set of the hydrated FFWMP samples with varying levels of the external standard mixture (13 compounds of interest prepared at 0.004% (*w/v*) in dH_2_O). Matrix (control) samples (FFWMP sample + dH_2_O only) were also included in each run. Samples were extracted for 45 min at a temperature of 43 °C. Each sample was subjected to a 10-min pre-extraction incubation time at 43 °C with pulsed agitation of 5 s at 500 rpm, automated by a Bruker CombiPal autosampler (Elementec Ltd., Maynooth, Co. Kildare, Ireland). Each sample was analysed in triplicate every two weeks during the 16 week storage period.

#### 2.7.2. GC Conditions

Incubation, extraction, and injection processes were implemented using a Bruker CombiPal autosampler (Elementec Ltd., Maynooth, Co. Kildare, Ireland). A mid-polar DB 624 UI column (60 m × 0.32 mm × 1.80 μm) (Agilent Technologies Ltd., Ireland) and a 2 cm, 50/30 μm, Divinylbenzene/Carboxen/Polydimethylsiloxane (DVB/CAR/PDMS) Stableflex SPME fibre (Agilent Technologies Ltd., Cork, Ireland) were used for the duration of the study. Following extraction, the SPME fibre was retracted and injected into the split/splitless 1177 GC inlet for 5 min at 250 °C in split mode at a ratio of 10:1 followed by 2 min at 270 °C in a bake-out station to minimise carry-over of compounds. The column oven was held at 65 °C for 10 min, then increased to 240 °C at a rate of 10 °C/min, and held for 4 min. Helium was used as the carrier gas with a constant flow rate of 1.0 mL/min.

### 2.8. Sensory Analysis

Powder samples were reconstituted in dH_2_O based on the fat and protein content as per the equation outlined by the International Dairy Federation (IDF) [24], to a total volume of 1.5 L 24 h prior to scoring and chilled 4 °C. Samples were allowed to equilibrate to room temperature before sampling commenced. Acceptance testing (hedonics) and RDA were carried out on the samples every four weeks for 16 weeks by approximately 18 semi-trained panellists familiar with scoring dairy products at University College Cork, Ireland. Each assessor was presented with 12 reconstituted milk powder samples labelled with random three-digit codes and was given as much time as he/she required to complete the scoring. Unlimited water was provided to each panellist.

### 2.9. Statistical Analysis

Statistical analysis for data relating to colour, composition, and microbial analysis was carried out using one-way ANOVA with post-hoc Tukey tests using the Statistical Package for the Social Sciences (SPSS) software, Version 24 (IBM Statistics Inc., Armonk, NY, USA). Pearson correlation analysis was carried out on the sensory and volatile data using SPSS. Principal component analysis (PCA) biplots of the volatile vs. sensory data were used to demonstrate correlations between the volatile compounds and the sensory attributes. These were constructed using the “factoextra” and “FactoMinoR” packages in R (v 3.4.1) [25].

## 3. Results and Discussion

### 3.1. General Powder Characteristics

Compositional results for the 12 reconstituted milk powder samples (10% total solids for SMP; 13% for FFWMP and IMF) are outlined in Table S1. The fat, protein, true protein, and casein content varied significantly (*p* ≤ 0.05) between the powders. As expected, the FFWMP contained the highest fat content (3.77%) and the SMP the lowest (0.02%). The IMF contained the highest level of lactose across all the storage conditions (7.53–7.89%). True protein and casein values were higher in SMP (3.64 and 2.89, respectively) than FFWMP and IMF.

No significant differences were observed between the total bacterial counts for the 12 reconstituted milk powder samples (4× FFWMP, 4× SMP, and 4× IMF) over the 16 week storage period (data not shown). Total bacteria counts were below the limit for powdered milk and milk based products intended for human consumption within Europe at all-time points [26].

### 3.2. Milk Powder Colour Analysis

The L* (lightness), a* (green-to-red colour), and b* (blue-to-yellow colour) values were statistically different (*p* ≤ 0.001) between the samples (Table S2). The L* and b* values were significantly (*p* ≤ 0.001) higher in FFWMP samples than in SMP and IMF samples regardless of the storage treatments. The a* values were negative across all samples with significantly (*p* ≤ 0.001) higher values observed for SMP than FFWMP and IMF samples across all treatments with the highest a* value observed in the SMP stored at 37 °C (–5.83). As expected, significantly (*p* ≤ 0.001) higher b* (yellowness) values were observed in FFWMP samples, due to the increased fat content [27]. Increased b* values (blue-to-yellow colour) in dairy products as observed in the FFWMP powder samples have previously been associated with β-carotene content [18], which has been linked with dairy products produced from pasture [28]. The significantly (*p* ≤ 0.001) higher a* value (green-to-red colour) observed in SMP samples analysed in this study could be due to the Tyndall effect; the scattering of light as it passes through a colloid. The higher casein content in SMP (2.79% compared with 2.31% and 1.07% for FFWMP and IMF, respectively) scattered more blue light than red. Furthermore, β-carotene is lost when milk is skimmed, removing the source of the yellow colour observed in FFWMP and IMF samples [29]. The L* (lightness) values were also significantly (*p* ≤ 0.001) different between the samples. Several other factors such as the modification of particle size, Maillard reactions forming brownish pigments during heat treatment [30], storage time, and temperature could all have contributed to the differences in colour.

### 3.3. Fatty Acid Analysis

Significant differences were observed for 19 of the 27 FA analysed based on sample type (C4:0, C6:0, C10:0, C12:0, C13:0, C14:0, C14:1 c9, C15:0, C16:0, C17:0, C18:1 n9c, C18:2 n6c, C18:2 n6t, C18:3 n3, C20:0, C20:1, C24:1 n9, C20:5, and CLA C18:2 c9t11), as shown in Table 1.

The FA composition of milk is generally derived from two primary sources, *via* uptake of existing FA from the diet (and rumen bio-hydrogenation) or through de novo synthesis [31]. C4:0-C14:0 and some C16:0 are synthesised de novo by the cow’s mammary gland, which uses acetate and ß-hydroxybutyrate as substrates [31]. The remaining C16:0 and the long-chain FA originate from dietary lipids and from lipolysis of adipose tissue triacylglycerols [32]. Bovine diet has been shown to impact the FA profile of milk significantly [33,34], and subsequently that of milk powder. The FA with an odd number of carbons such as pentadecanoic acid (C15:0) and heptadecanoic acid (C17:0) are synthesised by microflora in the rumen [35]. The FA profile of the FFWMP and the IMF varied more than the SMP. Less FA was detected in SMP due to the low fat content. FFWMP contained significantly (*p* ≤ 0.05) higher proportions of palmitic acid (C16:0) than the SMP and IMF samples. The IMF sample contained higher proportions of linoleic acid (C18:2 n6c) and α-linolenic acid (C18:3 n3) than the FFWMP, likely due to fortification. The FA C20:2, C20:3 n6, C24:1 n9, and C20:5 were identified only in the IMF sample. Some of these FA are found in fish oils [36] and vegetable oils [37,38], and thus, these were the likely sources in the IMF sample and were added during processing. The probable FA sources for the 13 compounds of interest are outlined in Table 2.

### 3.4. HS-SPME-GCMS Volatile Analysis

#### 3.4.1. FFWMP 

Volatile analysis was carried out on each of the milk powder samples by HS-SPME GCMS every two weeks (T0, T2, T4, T6, T8, T10, T12, T14, and T16) over the 16 week storage period. The 13 selected volatile aromatic compounds were quantified at each time point (Table 3). As expected, the highest levels of primary oxidation products were observed in FFWMP at T16 for ACC. Significant differences were observed in the concentrations of ten of the LO compounds in samples stored in ACC, nine stored in CON, seven stored in AM, and ten stored in HUM.

#### 3.4.2. SMP

Much less variability in the levels of aldehyde and ketone compounds was observed in the SMP samples (Table 3). However, the alcohol compounds, 1-heptanol and 1-pentanol, were more unstable in the SMP samples. As with the FFWMP samples, more compounds (ten) varied significantly in the SMP samples stored at ACC than samples stored at CON (two), AM (three), and HUM (five).

#### 3.4.3. IMF

When compared to FFWMP and SMP, increased levels of some oxidation products were observed in IMF samples at T0 and throughout the study at all-time points and in all storage conditions (CON, AM, ACC, and HUM) (Table 3). Hexanal, pentanal, heptanal, octanal, 2,4-decadienal, and 2-nonanone were present at 5986, 1209, 861, 784, 154, and 5170 ppm, respectively, at T0. The highest levels LO volatiles were observed at T12 and T16 in IMF samples stored in ACC (similar to the trend for the FFWMP samples). The concentrations of 11 target compounds varied significantly during ACC storage; 11 compounds varied significantly in CON samples, seven in AM samples, and 11 in HUM samples. Concentration (mg/kg of powder) results for each volatile compound are outlined in Table 3.

The concentrations of the aldehydes hexanal, pentanal, and heptanal increased throughout storage in the both the FFWMP and IMF samples stored in ACC (3.77 and 2.91% fat, respectively). The sensory attributes associated with these compounds have been described as “painty”, “cardboard-like”, and “grassy” [17,18]. Hexanal and 2-heptanone have been found to be good predictors of “grassy flavour” in previous studies, while hexanal, octanal, and 3-octen-2-one have been found to be good predictors of “painty flavour” in WMP [6]. Hexanal has also been identified as the main contributor to the “oxidised flavour” often observed in dairy powders as LO progresses [42]. Increased concentrations of octanal and 3-octen-2-one were observed in IMF samples compared to FFWMP and SMP samples. Li et al. [42] reported higher levels of octanal and 3-octen-2-one in concentrated milk and milk powders than in raw and heated milk, suggesting that these compounds are likely thermally induced LO products. Park and Drake [43] also reported increases in octanal in liquid condensed milk after 24 h storage, indicative of LO. However, in the present study, the levels of octanal fluctuated throughout storage in the IMF samples, and the lowest levels were observed in ACC samples (142 ppm ± 5.32). (E)-2-Nonenal, 2,4-decadienal, and 1-heptanol were found to be higher in IMF samples, particularly in the samples stored at ACC. The concentrations of some other volatile compounds fluctuated and/or decreased during storage, possibly also due to conversion and breakdown to other compounds that were not quantified in this study.

### 3.5. Sensory Evaluation

**Figure 1:**
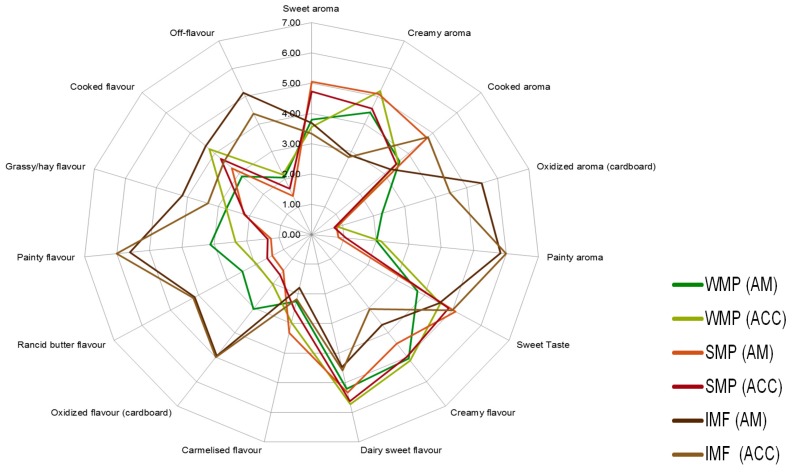
Radar plot illustrating the ranked descriptive analysis (RDA) scores for the sensory attributes evaluated at T16 for samples stored at 21 °C (AM) and 37 °C (ACC).

No significant differences were observed between the FFWMP or SMP samples for hedonic assessment over the storage period. Significant differences were found between “liking of flavour” and “overall acceptability” for the IMF samples. The IMF samples scored lowest for “overall acceptability” across all time points and storage treatments. At T4, significant differences were observed between the FFWMP, SMP, and IMF samples for “liking of appearance”, “liking of aroma”, “liking of flavour”, “overall acceptability”, “colour”, “creamy aroma”, “oxidised aroma”, “painty aroma”, “powdery texture”, “oxidised flavour”, and “off-flavour”. Again, at T8, differences between the samples were observed for “liking of appearance”, “liking of aroma”, “liking of flavour”, and “overall acceptability”, with IMF samples scoring the lowest for “liking of aroma”, “liking of flavour”, and “overall acceptability” and highest for “liking of appearance”. SMP samples scored highest for “liking of aroma”, “liking of flavour”, and “overall acceptability”. In the RDA, differences were again observed for “oxidised aroma”, “painty aroma”, “powdery texture”, and “off-flavour” in addition to “rancid butter flavour” and “painty flavour”, all of which were more correlated with IMF samples except for “powdery texture”, which was more correlated with FFWMP samples. Finally, at T16, significant differences were observed for “creamy aroma”, “oxidised aroma”, “painty aroma”, “painty flavour”, and “off-flavour” (Figure 1). This study demonstrated the ability of panellists to identify and rate the intensity of “painty” and “oxidised” attributes in powders with high levels of LO volatiles. When the samples were compared based on treatment type, fewer differences were observed, suggesting the differences were based on sample type regardless of the storage conditions.

**Figure 2:**
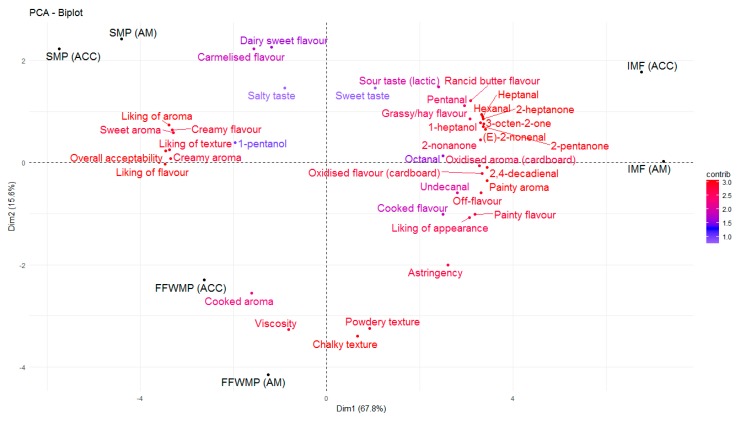
Principle component analysis (PCA) biplot of fat-filled whole milk powder (FFWMP), skim milk powder (SMP), and infant milk formula (IMF) samples at T8 demonstrating the trends observed throughout the study for the ranked descriptive analysis (RDA) and volatile analyses. AM: ambient, 21 °C; ACC: accelerated, 37 °C.

**Figure 3:**
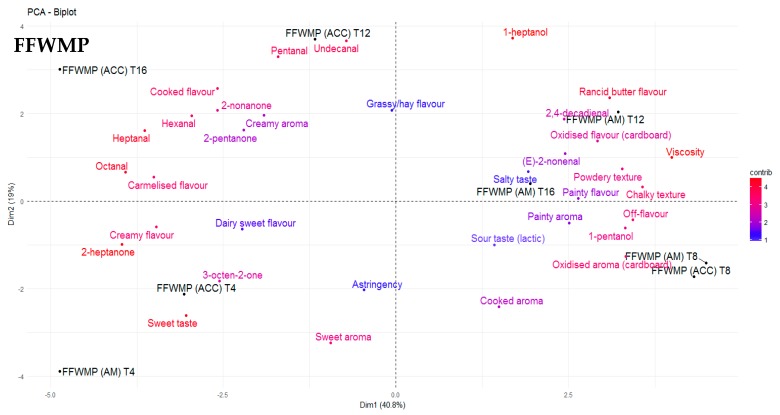
Principle component analysis (PCA) biplot demonstrating the correlations between the sensory perception and volatile compounds for fat-filled whole milk powder (FFWMP) at T4, T8, T12, and T16 of storage. AM: ambient, 21 °C; ACC: accelerated, 37 °C.

**Figure 4:**
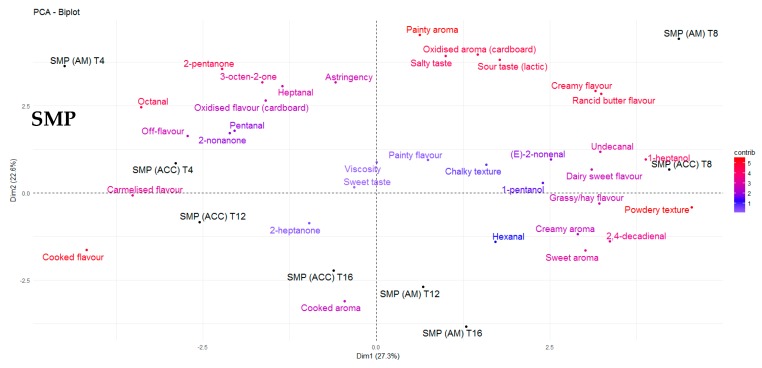
PCA biplot demonstrating the correlations between the sensory perception and volatile compounds for skim milk powder (SMP) at T4, T8, T12, and T16 of storage. AM: ambient, 21 °C; ACC: accelerated, 37 °C.

**Figure 5:**
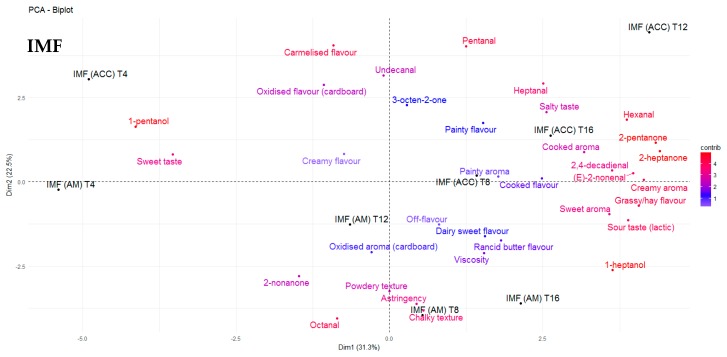
Principle component analysis (PCA) biplot demonstrating the correlations between the sensory perception and volatile compounds for infant milk formula (IMF) samples at T4, T8, T12, and T16 of storage. AM: ambient, 21 °C; ACC: accelerated, 37 °C.

The odour threshold of many volatile compounds is greater in oil and fat matrices when compared with water and air, generally due to the complexity of the matrix and possible matrix binding [44]. In the present study, FFWMP samples contained hexanal at 380 ppm and pentanal at 56 ppm, but remained acceptable to the sensory panellists. Decker et al. [40] reported an odour threshold (ppm) in oil of 320, 240, 55, and 10 for hexanal, pentanal, octanal, and 2,4–decadienal, respectively. The increased levels of numerous compounds above their odour thresholds in IMF samples compared to FFWMP and SMP samples was likely responsible for the unacceptable scores of panellists for these IMF samples. Additionally, certain compounds such as (E)-2-octenal (linoleic acid degradation), (Z)-2-heptenal (linoleic acid degradation), (E)-2-hexenal (linolenic acid degradation), 2,4-heptadienal (linolenic acid degradation), 4-pentenal, and (E,E)- 3,5-octadien-2-one (arachidonic and linoleic acid degradation) were identified only in IMF samples. 2,4–Decadienal, an oxidation product of linoleic acid, has been described as having a “frying” or “fried” odour and is reported to have a pleasant association with high quality fried foods, and it is only when the concentrations are excessive that a product becomes unacceptable to the consumer [40], as the odour changes to a more rancid off-note. 2,4-Decadienal was found to be correlated with “rancid butter flavour” in FFWMP samples from T8 to T16, where the concentrations ranged from 15–61 ppm. However, 2,4–decadienal was also found to be correlated with “painty aroma” and “painty flavour”. Karahadian and Lindsay [45] reported that 2,4–decadienal can cause “painty flavours” in fish oils, and this may also apply for dairy powders once the concentrations reach a certain level. Furthermore, similar volatiles could have the same descriptors in different products. For example, 2,4-decadienal and undecanal could both be descriptors of “oxidised flavour” in FFWMP and IMF, respectively. 3-Octen-2-one was again highest in IMF samples followed by FFWMP and SMP samples. PCA analysis showed that 3-octen-2-one was correlated with “caramelised flavour” and “sweet taste” in FFWMP and “oxidised flavour” and “painty flavour” in IMF. Overall, relative humidity did not significantly affect the levels of any LO volatile compound as samples stored at HUM were comparable to those stored at AM. Most differences were observed between samples stored at AM and ACC; thus, for clarity, we only focussed on AM and ACC samples in Figure 2, Figure 3, Figure 4 and Figure 5.

Many significant differences were observed for the hedonic and RDA scores, particularly between SMP and IMF samples, but also within SMP and IMF samples stored in different conditions at different time points. FFWMP remained stable and acceptable throughout the 16 week storage period despite increases in LO volatile compounds. This suggested that although these compounds were present at levels above their odour thresholds, and thus potentially perceivable, they did not adversely impact sensory perception, presumably because they were not concentrated enough in the FFWMP matrix. SMP samples remained acceptable throughout storage, also scoring highest for “overall acceptability” across the storage treatments. The IMF samples were found to be unacceptable at each time point. 

Levels of the oxidation products hexanal, pentanal, and heptanal were present at 5986, 1209, and 861 ppm, respectively in IMF samples at T0 with significant increases over storage. As previously mentioned, the level of sensory acceptability for IMF samples remained the same from T4 to T16, which suggested that once LO products reached certain levels above their odour thresholds, panellists deemed the product as “unsatisfactory”. Many of the descriptors commonly used to describe off-flavours associated with LO in dairy products were most correlated with IMF samples (Figure 2). Correlations between sensory data and volatile profiles for FFWMP, SMP, and IMF are displayed in Figure 3, Figure 4 and Figure 5, respectively. “Painty”, “oxidised”, “rancid butter”, and “off-flavours” were most correlated with the IMF samples at T12 and T16, corresponding with increases in hexanal, heptanal, and pentanal (Figure 5).

The ability of the sensory panel to perceive differences in many sensory attributes in SMP was unexpected as it is typically less susceptible to LO due to its low-fat content. However, the lipid phase of WMP and other high-fat dairy powders could act as a solvent for LO compounds [46]; thus, the lack of fat in SMP could mean that any oxidation products were more readily released and therefore were more easily perceived. It is difficult to compare the sensory perception of IMF to that of FFWMP and SMP for a number of reasons: (i) the differences in manufacturing processes and the addition of PUFA to IMF samples; (ii) the adult panellists employed in the study were not familiar with the consumption of IMF; and (iii) it was impossible to gather information from the proposed consumers of IMF (infants and babies) on sensory perception. However, it is not unusual to see higher levels of LO products in IMF when compared to conventional milk powders. Cesa et al. [12] reported the levels of malondialdehyde (MDA), a common indicator of the LO process, up to five times higher in IMF compared to bovine milk samples. Furthermore, Jia et al. [13] conducted an accelerated stability study on milk based IMF stored at 42 and 50 °C for 90 days. Results demonstrated little change in the FA profile of the IMF during storage except for docosahexaenoic acid (C22:0). However, differences in the volatile profiles were observed, and an unpleasant “oxidised flavour” was observed in IMF samples stored at 50 °C. Samples stored at 50 °C were found to have increased peroxide values and decreased headspace oxygen after 90 days of storage. As little or no changes in the FA profile were evident, it suggested that the susceptibility of IMF to LO depended mainly on the FA composition directly after manufacture and subsequently on the rate at which the FA oxidised.

Correlation relationships were observed between volatile compounds and sensory perception and between the concentrations of individual LO volatile compounds. The main correlation relationships observed in FFWMP samples are outlined in Table 4. Furthermore, “sweet taste” decreased as “rancid butter flavour” increased in the AM FFWMP samples throughout storage. Less correlation was evident in SMP samples; however, “cooked flavour” was negatively correlated with undecanal (–0.83). In the IMF samples, “grassy/hay flavour” was positively correlated with 1-heptanol (*r* = 0.74) and 1-pentanol (*r* = 0.74), and “off-flavours” were positively correlated with (E)-2-nonenal. “Overall acceptance” was negatively correlated with “oxidised aroma” (–0.53) in IMF samples, and there was no correlation between “overall acceptability” and sensory attributes in the FFWMP and SMP samples. Lloyd et al. [6] reported similar correlations for hexanal and heptanal and “painty flavour” and “grassy flavours” in WMP. That study [6] concluded that the optimum shelf-life of WMP was approximately 12 weeks from a sensory standpoint.

In the present study, freshly opened WMP and SMP remained acceptable to the sensory panel for the whole 16 weeks of storage despite levels of primary oxidation products being present above their odour thresholds. Storing samples in ACC versus AM accelerated the formation of LO compounds, which contradicted the results of Cesa et al. [12], which stated that 40 °C was too low a temperature to perform accelerated oxidation studies and that the levels of MDA in samples stored at 40 °C were comparable to those stored at 20 °C after 12 weeks of storage. The study by Cesa et al. [12] recommended a temperature of 55 °C for performing acceleration studies. However, in the present study, differences in the volatile profiles were evident in FFWMP and SMP samples stored in AM and ACC. MDA quantification also had limitations [47,48], as other compounds could interfere with the assay and also that it was specific for only one LO chemical class. Therefore, accurate quantification of LO volatile compounds by HS-SPME GCMS as preformed in this study is a much more reliable indicator of powder quality.

### 3.6. Range of Lipid Oxidation in Six Retail Infant Milk Formula Products

The amount of total fat as outlined by the manufacturers did not vary significantly between the IMF retail brands (Table 5); however, the FA profile, including the levels of PUFA, did (Table S3).

The levels of the aldehydes hexanal, pentanal, heptanal, and octanal were significantly (*p* ≤ 0.001) higher in Brand 1 compared to the other four brands of IMF powdered samples (Brand 2–5); the same was observed for the alcohol compounds 1-heptanol and 1-pentanol. *Trans*-2-nonenal and 2,4–decadienal were significantly higher in Brands 3, 4, and 5 when compared to Brands 1 and 2. 2-Heptanone was significantly higher in Brand 2 compared with Brands 1, 3, 4, and 5. Levels of undecanal, 2-nonanone, 2-pentanone, and 3-octen-2-one did not vary significantly between the samples (Table 6). The ultra-heat treated (UHT) ready-made IMF (Brand 6) contained increased levels of pentanal, hexanal, heptanal, and 2-heptanone present at 4549, 317, 269, and 174 ppm, respectively. The other nine LO compounds of interest (octanal, undecanal, (E)-2-nonenal, 2,4-decadienal, 2-penanone, 2-nonanone, 3-octen-2-one, 1-heptanol, 1-pentanol) were not detected. A total of 25 FA were quantified in the IMF samples, 20 of which varied significantly.

Brand 5 contained the highest percentage of palmitic acid (C16:0) (24.36 ± 1.72) and oleic acid (C18:1) n9c (30.36 ± 1.52). Brands 2, 3, and 4 contained significantly higher proportions of lauric acid (C12:0) compared to Brands 1 and 5. The total FA contents of IMF Brands 1–5 are shown in Table S3.

The increased concentrations of certain volatile compounds observed in the six retail IMF samples likely related to the significant differences in FA profile. The FA potentially originating from the addition of fish and vegetable oils (C20:2, C20:3 n6, C24:1 n9, and C20:5) were likely some of the main contributors to the observed oxidative state of the IMF powders. The significantly higher levels of primary aldehyde and secondary alcohol compounds in Brand 1 compared to the other brands indicated issues in relation to the fat component of this product, which resulted in more LO products present immediately after manufacture. The level of long-chain PUFA was slightly higher in Brand 1, however unlikely to be responsible for the significantly higher levels of short- and medium-chain aldehydes. The compounds (E,E)–2,4-heptadienal, (E,E)–2,4-nonadienal (derived from linolenic and linoleic acid degradation), and 2-pentylfuran (derived from linoleic acid degradation) were only identified in Brand 1. These compounds have been shown to be high-impact flavour compounds in edible oils with relative odour activity values ≥ 1 [49]. Compounds with relative odour activity values ≥ 1 significantly contribute to aroma and are considered key volatile components. Brand 6, the UHT ready-made IMF, contained less oxidation compounds than the powdered products (Brands 1–5). UHT is used in the dairy industry as a means of preparing milk for long-term storage without the need for refrigeration. Ajmal et al. [50] found that the level of short-, medium-, and long-chain FA decreased in UHT milk compared to raw milk, and the FA profile of UHT milk remained stable during 30 days of storage. However, studies have reported Maillard reaction products present in milk post UHT processing [51], and “sulphury flavours” in milk have also been reported as a result of UHT processing [52]. Dimethyl disulfide and dimethyl trisulfide were also identified in Brand 6 in this study.

The antioxidant capacity of dairy powders is mainly determined by the levels of sulphur containing amino acids such as cysteine, but also levels of phosphate, carotenoids, zinc, selenium, and vitamins A and E [53]. Depending on their concentration and polarity, the natural occurrence of these antioxidants in milk, as well as supplementation during processing play a role in the rate at which LO progresses. Antioxidants work by scavenging free radicals and donating hydrogen, potentially slowing the LO cascade mechanism [54]. However, the levels of antioxidants were comparable across all brands of IMF, which provided further evidence that processing conditions were the main factor behind the rate of LO observed, especially evident for Brand 1. IMF is generally produced by wet mixing, dry blending, or a combination of both [55]. Studies have shown that the oxidative stability of IMF is influenced by the quality of the blended emulsion prior to spray drying and that un-homogenised emulsions can result in higher levels of free fat in the final product [56]. In addition to antioxidant addition, encapsulation of PUFA has been employed as a secondary approach to protect them against oxidation in IMF [57]. Some of the materials available for carriers of PUFA and other lipids are plant polysaccharides, proteins, and peptides [58]. The quality and source of the milk from which the IMF is produced is also likely to impact the LO susceptibility of the final product as bovine diet has also been proven to impact the milk’s FA profile [33,34].

## 4. Conclusions

The FA profile varied significantly between the samples and some long-chain FA were identified only in IMF samples (C20:2, C20:3 n6, C24:1 n9, and C20:5). The likely source for these FA in the IMF was fortification with vegetable and fish oils during manufacture followed by thermal processing, which could initiate the LO process, resulting in increased susceptibility to LO. The volatile profile of the FFWMP, SMP, and IMP also varied significantly, which was related to differences in FA profile, and possibly due to the origin of the milk, i.e., bovine diet and presence or absence of pro- and anti-oxidants. Overall, increases in the concentrations of hexanal, heptanal, and pentanal were good indicators of LO occurring in FFWMP. Fewer significant increases in LO compounds were evident in SMP; however, changes in the concentrations of hexanal, 1-heptanol, and 1-pentanol were evident. Regarding IMF, significant increases in specific aldehydes hexanal, pentanal, heptanal, and octanal were good indicators of LO. The concentrations of (E)-2-nonenal and 2,4-decadienal were also higher in IMF samples compared to FFWMP and SMP. The sensory acceptance scores for FFWMP and SMP remained stable throughout storage, despite some LO compounds being perceived by the panellists. The IMF sample was perceived negatively from the start of storage due to high levels of numerous LO compounds present. “Oxidised” and “painty” attributes were correlated with increased concentrations of hexanal and heptanal and were particularly evident in IMF samples. In addition to the main experiment, the FA content and volatile profile of six widely available IMF brands (five powdered and one ready-to-use UHT product) were evaluated. The proportions of 20 of the 25 FA identified varied significantly between the powdered IMF brands, while the concentrations of nine of the 13 volatile compounds also varied significantly. The concentrations of volatile compounds, in particular aldehydes, were also very high in the six IMF brands in comparison to the FFWMP and SMP samples.

## Figures and Tables

**Table 1:** Fatty acid (FA) composition (g/100 g of FA ± SD; *n* = 3) of fat-filled whole milk powder (FFWMP), skim milk powder (SMP), and infant milk formula (IMF). Different superscripts within a row indicate significant differences between the samples (*p* = 0.05).

Fatty Acids	FFWMP	SMP	IMF	*p*-Value
Butyric acid C4:0	0.09 ± 0.02 ^b^	21.03 ± 5.93 ^a^	1.03 ± 0.07 ^b^	0.014
Caproic acid C6:0	0.04 ± 0.01 ^b^	6.64 ± 2.02 ^a^	0.41 ± 0.01 ^b^	0.018
Octanoic acid C8:0	0.04 ± 0.01	1.32 ± 1.86	0.44 ± 0.01	0.549
Decanoic acid C10:0	0.04 ± 0.01 ^b^	3.98 ± 0.28 ^a^	0.65 ± 0.03 ^b^	<0.001
Undecanoic acid C11:0	ND	ND	0.01 ± 0.01	0.465
Lauric acid C12:0	0.28 ± 0.02 ^b^	4.40 ± 0.83 ^a^	3.03 ± 0.15 ^ac^	0.008
Tridecanoic acid C13:0	ND ^b^	ND ^b^	0.02 ± 0.01 ^a^	<0.001
Myristic acid C14:0	0.49 ± 0.29 ^b^	ND ^b^	2.43 ± 0.08 ^a^	0.002
Myristoleic acid C14:1 c9	0.01 ± 0.01 ^b^	ND ^b^	0.13 ± 0.01 ^a^	0.001
Pentadecanoic acid C15:0	0.06 ± 0.01 ^b^	ND ^c^	0.23 ± 0.01 ^a^	<0.001
Palmitic acid C16:0	32.9 ± 3.71 ^a^	17.18 ± 3.71 ^b,c^	20.06 ± 0.21 ^b^	0.027
Palmitoleic acid C16:1 c9	0.13 ± 0.01	0.88 ±0.37	0.26 ± 0.01	0.075
Heptadecanoic acid C17:0	0.08 ± 0.01 ^b^	ND ^c^	0.14 ± 0.01 ^a^	0.001
Stearic acid C18:0	3.36 ± 0.37	1.22 ± 1.72	4.31 ± 0.07	0.114
Oleic acid C18:1 n9c	29.22 ± 2.89 ^a^	10.59 ± 6.74 ^b^	26.77 ± 0.45 ^a,b^	0.040
Elaidic acid C18:1 n9t	2.59 ± 0.36	2.01 ± 2.85	2.92 ± 0.03	0.863
Linoleic acid C18:2 n6c	8.40 ± 3.19 ^b^	30.76 ± 2.99 ^a^	17.32 ± 0.24 ^b,c^	0.007
*trans*-9,12-octadecadienoate C18:2 n6t	21.60 ± 3.25 ^a^	ND ^b^	15.91 ± 0.28 ^a^	0.003
α-Linolenic acid C18:3 n3	0.17 ± 0.02 ^b^	ND ^c^	1.79 ± 0.04 ^a^	<0.001
Gamma linolenic acid c18:3 n6	0.01 ± 0.01	ND	0.07 ± 0.01	0.151
Eicosanoic acid C20:0	0.24 ± 0.03 ^a^	ND ^b^	0.24 ± 0.01 ^a^	0.001
*cis*-11-Eicosenoic acid C20:1	0.10 ± 0.01 ^b^	ND ^c^	0.24 ± 0.01 ^a^	<0.001
Eicosenoic acid C20:2	ND	ND	0.01 ± 0.02	0.465
Eicosadienoic acid C20:3 n6	ND	ND	0.01 ± 0.02	0.465
Nervonic acid C24:1 n9	ND ^b^	ND ^b^	0.28 ± 0.05^a^	0.004
Eicosapentaenoic acid C20:5	ND ^b^	ND ^b^	0.05 ± 0.05 ^a^	<0.001
CLA C18:2 c9t11	0.14 ± 0.03 ^b^	ND ^c^	1.28 ± 0.01 ^a^	<0.001

CLA: conjugated linoleic acid; ND: not detected; c: *cis*; t: *trans*.

**Table 2:** The probable fatty acid sources for the 13 volatile compounds of interest.

Class	Compound	Fatty Acid Source	Reference
Aldehyde	Hexanal	Oleic and linoleic acid	[17]
	Pentanal	Arachidonic and linoleic acid	[17]
	Heptanal	Possibly linoleic and oleic acid	[39]
	Octanal	Oleic acid	[40]
	(E)-2-Nonenal	Linoleic and possibly palmitoleic acid	[40]
	2,4-Decadienal	Linoleic acid	[40]
	Undecanal	Possibly oleic acid	-
Ketone	2-Nonanone	Decanoic acid	[41]
	2-Heptanone	Octanoic acid	[41]
	2-Pentanone	Hexanoic acid	[41]
	3-Octen-2-one	Arachidonic and linoleic acid	[17]
Alcohol	1-Heptanol	Possibly lipid oxidation of heptanal	-
	1-Pentanol	Lipid oxidation of pentanal	[17]

**Table 3:** Concentrations (mg/kg of powder; *n* = 3) of the 13 volatile compounds in the fat-filled whole milk powder (FFWMP), skim milk powder (SMP), and infant milk formula (IMF) samples at each time point (T0, T4, T8, T12, and T16). CON: control, −18 °C; AM: ambient, 21 °C; ACC: accelerated, 37 °C; HUM: humidity, 25 °C and 50% humidity. * *p* = 0.05, *** *p* = 0.001.

**Compound**	**LRI**	**T0 FFWMP**	**T4 FFWMP (CON)**	**T8 FFWMP (CON)**	**T12 FFWMP (CON)**	**T16 FFWMP (CON)**	***p*-Value**	**T4 FFWMP (AM)**	**T8 FFWMP (AM)**	**T12 FFWMP (AM)**	**T16 FFWMP (AM)**	***p*-Value**	**T4 FFWMP (ACC)**	**T8 FFWMP (ACC)**	**T12 FFWMP (ACC)**	**T16 FFWMP (ACC)**	***p*-Value**	**T4 FFWMP (HUM)**	**T8 FFWMP (HUM)**	**T12 FFWMP (HUM)**	**T16 FFWMP (HUM)**	***p*-Value**
**Hexanal**	840	0	277	197	133	82	*	193	145	132	118	***	259	189	324	382	***	217	207	174	124	*
**Pentanal**	735	0	11	9	13	6	*	10	8	14	10	***	13	13	56	41	***	9	12	18	16	*
**Heptanal**	944	15	42	17	15	20	*	35	22	19	23	**NS**	31	20	40	50	***	24	20	26	25	*
**Octanal**	1047	22	33	17	18	21	*	36	15	21	12	**NS**	28	12	30	37	***	30	25	23	28	*
**(E)-2-Nonenal**	1151	14	5	9	10	9	*	6	11	16	12	**NS**	4	4	6	5	***	12	9	9	11	*
**2,4-Decadienal**	1399	37	2	15	20	17	*	6	33	61	54	**NS**	8	15	20	22	***	6	53	31	29	*
**Undecanal**	1359	7	3	5	3	2	*	4	7	13	9	***	4	4	32	17	***	19	34	17	14	*
**2-Nonanone**	1140	0	17	10	82	66	*	21	12	3	2	***	5	1	30	109	***	5	4	36	92	**NS**
**2-Heptanone**	935	10	9	6	5	6	**NS**	28	7	7	6	**NS**	19	6	16	19	***	22	6	8	8	**NS**
**2-Pentanone**	730	2	1	3	3	1	**NS**	2	1	1	0	***	1	2	2	10	**NS**	2	1	1	5	*
**3-Octen-2-one**	1096	20	21	10	4	19	**NS**	24	9	5	7	***	6	2	7	9	***	6	6	3	3	*
**1-Heptanol**	1016	0	0	41	19	31	*	0	36	50	32	***	0	29	51	46	**NS**	0	55	48	38	*
**1-Pentanol**	815	70	60	329	136	189	**NS**	54	274	311	17	**NS**	48	365	39	48	**NS**	74	35	124	1793	**NS**
**Compound**	**LRI**	**T0 SMP**	**T4 SMP (CON)**	**T8 SMP (CON)**	**T12 SMP (CON)**	**T16 SMP (CON)**	***p*-Value**	**T4 SMP (AM)**	**T8 SMP (AM)**	**T12 SMP (AM)**	**T16 SMP (AM)**	***p*-Value**	**T4 SMP (ACC)**	**T8 SMP (ACC)**	**T12 SMP (ACC)**	**T16 SMP (ACC)**	***p*-Value**	**T4 SMP (HUM)**	**T8 SMP (HUM)**	**T12 SMP (HUM)**	**T16 SMP (HUM)**	***p*-Value**
**Hexanal**	840	0	14	42	28	20	**NS**	2	4	29	11	*	14	48	29	10	*	8	42	19	8	*
**Pentanal**	735	3	2	1	2	1	**NS**	7	1	2	2	**NS**	2	4	1	1	*	3	3	1	1	**NS**
**Heptanal**	944	16	10	16	5	7	**NS**	16	13	6	8	**NS**	8	4	5	9	*	16	15	1	3	*
**Octanal**	1047	18	12	6	13	17	*	17	7	9	4	**NS**	10	5	7	7	*	10	9	10	12	**NS**
**(E)-2-Nonenal**	1151	20	2	4	2	2	**NS**	2	5	4	3	**NS**	2	2	1	2	*	3	2	2	3	**NS**
**2,4-Decadienal**	1399	15	1	6	5	5	**NS**	1	8	8	7	**NS**	1	4	4	4	*	1	8	5	6	**NS**
**Undecanal**	1359	5	1	2	1	2	**NS**	1	4	3	2	**NS**	1	2	1	1	*	2	2	2	1	**NS**
**2-Nonanone**	1140	7	6	5	3	3	*	7	5	4	3	*	4	4	6	8	*	4	3	4	4	*
**2-Heptanone**	935	9	6	7	3	3	**NS**	7	5	6	4	**NS**	4	5	7	13	*	5	5	7	9	**NS**
**2-Pentanone**	730	1	1	0	0	0	**NS**	1	0	0	0	**NS**	1	0	0	0	**NS**	1	0	0	0	**NS**
**3-Octen-2-one**	1096	4	4	2	1	1	**NS**	5	3	0	2	**NS**	2	1	0	1	*	1	6	1	1	*
**1-Heptanol**	1016	37	0	55	61	49	**NS**	0	61	16	22	**NS**	0	46	35	24	**NS**	0	41	43	53	*
**1-Pentanol**	815	0	61	83	4710	82	**NS**	63	32	123	47	*	62	1014	82	50	**NS**	56	105	47	51	**NS**
**Compound**	**LRI**	**T0 IMF**	**T4 IMF (CON)**	**T8 IMF (CON)**	**T12 IMF (CON)**	**T16 IMF (CON)**	***p*-Value**	**T4 IMF (AM)**	**T8 IMF (AM)**	**T12 IMF (AM)**	**T16 IMF (AM)**	***p*-Value**	**T4 IMF (ACC)**	**T8 IMF (ACC)**	**T12 IMF (ACC)**	**T16 IMF (ACC)**	***p*-Value**	**T4 IMF (HUM)**	**T8 IMF (HUM)**	**T12 IMF (HUM)**	**T16 IMF (HUM)**	***p*-Value**
**Hexanal**	840	5986	10674	13408	11700	13408	*	14364	17047	14581	17047	*	15628	19874	20741	19874	*	12550	18947	14353	15659	*
**Pentanal**	735	1209	1200	1111	1520	1111	*	1367	1530	2006	1530	*	3934	3621	3999	3621	*	1348	1401	1628	1641	*
**Heptanal**	944	861	915	1080	915	1080	*	1310	1313	1143	1313	*	1322	1410	1588	1410	*	1095	1399	1055	1295	*
**Octanal**	1047	784	608	864	803	864	*	910	1095	1039	1095	**NS**	133	146	141	146	*	799	1370	841	1090	**NS**
**(E)-2-Nonenal**	1151	64	36	53	60	53	*	46	79	93	79	*	48	74	110	74	*	55	86	132	97	*
**2,4-Decadienal**	1399	154	25	33	60	33	*	17	82	120	82	**NS**	39	72	136	72	*	37	82	148	122	*
**Undecanal**	1359	97	3	6	3	6	**NS**	6	18	11	18	**NS**	69	7	68	7	*	32	5	16	5	*
**2-Nonanone**	1140	5170	31	30	23	30	*	53	44	29	44	*	29	32	30	32	*	7064	7048	3871	3066	*
**2-Heptanone**	935	55	49	44	48	44	*	53	66	57	66	**NS**	51	69	81	69	*	49	49	48	57	*
**2-Pentanone**	730	8	10	9	9	9	*	7	13	11	13	*	11	15	16	15	*	8	9	14	15	*
**3-Octen-2-one**	1096	38	76	67	155	67	*	171	166	32	127	**NS**	174	147	218	147	**NS**	25	26	96	95	*
**1-Heptanol**	1016	0	0	1485	1309	1485	**NS**	0	2341	888	2341	**NS**	0	1731	1419	1191	**NS**	0	2201	1656	1116	**NS**
**1-Pentanol**	815	62	534	68	85	68	*	685	112	69	112	*	848	102	129	102	*	1250	107	85	85	*

LRI: linear retention index. NS: not significant.

**Table 4:** Correlation relationships between volatile organic compounds (VOCs) and sensory attributes observed in fat-filled whole milk powder (FFWMP). Positive and negative r values indicate significant (*p* ≤ 0.001) positive and negative correlations between the VOCs and sensory attributes, respectively.

Volatile Organic Compound	Hexanal	Rancid Butter Flavour	Grassy/Hay Flavour	Painty Flavour	Painty Aroma	Oxidised Flavour	Oxidised Aroma	Creamy Flavour	Creamy Aroma
Hexanal	-	0.87	0.82	0.89	0.92	0.92	0.88	–0.80	–0.81
Pentanal	0.91	-	-	0.81	0.80	0.83	-	-	-
Heptanal	0.99	0.87	0.81	0.89	0.93	0.92	0.89	–0.80	–0.83
(E)-2-Nonenal	0.93	0.83	0.85	-	0.91	0.90	0.84	–0.84	-
Octanal	-	-	-	-	0.80	-	-	-	-
2,4-Decadienal	-	-	-	-		0.80	-	-	-
2-Heptanone	-	0.82	0.80	-	0.90	0.88	-	-	–0.81
2-Pentanone	-	0.84	0.84	-	0.91	0.88	-	-	-
1-Heptanol	-	-	0.81	-	-	-	-	-	-

**Table 5:** Amount (g)/100 mL of prepared infant milk formula as stated on the product packaging of Brands 1–6.

Typical Values (g)	Brand 1	Brand 2	Brand 3	Brand 4	Brand 5	Brand 6
Protein	1.6	1.25	1.3	1.3	1.25	1.3
Fat	3.3	3.6	3.4	3.4	3.5	3.4
saturated	1.1	1.5	1.5	1.5	1.2	1.5
unsaturated	1.8	2.1	1.9	1.9	0.7	1.9
LCPs (not specified)	0.028	-	0.015	0.024	0.02	0.024
Linoleic acid	-	0.55	-	-	0.6	-
α-linolenic acid	-	0.067	-	-	0.07	-
Arachidonic acid (AA)	0.012	0.0084	0.006	0.011	0.012	0.011
Docosahexaenoic acid (DHA)	0.011	0.0084	0.006	0.01	0.007	0.01
Vegetable oils	Y	Y	Y	Y	Y	Y
Fish oils	Y	Y	Y	Y	Y	Y

LCP: long-chain polyunsaturated fatty acid. Y: yes.

**Table 6:** Concentrations (mg/kg of powder; *n* = 3) of the 13 volatile compounds quantified in the five infant milk formula (IMF) powder samples (Brands 1-5) purchased from local retail units. Different superscripts within a row indicate statistical differences when *p* = 0.001.

Class	Volatile Organic Compound	CAS No.	LRI	IMF Brand 1	IMF Brand 2	IMF Brand 3	IMF Brand 4	IMF Brand 5	*p*-Value
**Aldehyde**	**Hexanal**	66-25-1	840	17935 ^a^	488 ^b^	175 ^b^	285 ^b^	849 ^b^	*
	**Pentanal**	110-62-3	735	2013 ^a^	10 ^b^	15 ^b^	28 ^b^	51 ^b^	*
	**Heptanal**	111-71-7	944	1401 ^a^	73 ^b^	53 ^b^	52 ^b^	109 ^b^	*
	**Octanal**	124-13-0	1047	1815 ^a^	99 ^b^	162 ^b^	142 ^b^	212 ^b^	*
	**(E)-2-Nonenal**	18829-56-6	1151	662 ^a,d^	100 ^b,c^	1304 ^c,d^	1017 ^c^	1119 ^d^	*
	**2,4-Decadienal**	2363-88-4	1399	651 ^a^	1987 ^a^	14869 ^b^	13685 ^b^	16276 ^b^	*
	**Undecanal**	112-44-7	1359	1078	938	1229	984	405	NS
**Ketone**	**2-Nonanone**	821-55-6	1140	112	17	100	109	132	NS
	**2-Heptanone**	110-43-0	935	83 ^b^	2395 ^a^	22 ^b^	37 ^b^	41 ^b^	*
	**2-Pentanone**	107-87-9	730	18	10	4	40	33	NS
	**3-Octen-2-one**	1669-44-9	1096	1004	712	414	554	169	NS
**Alcohol**	**1-Heptanol**	111-70-6	1016	1177 ^a^	104 ^b^	176 ^b^	164 ^b^	271 ^b^	*
	**1-Pentanol**	71-41-0	815	2402 ^a^	538 ^b^	59 ^b^	93 ^b^	284 ^b^	*

CAS No.: Chemical Abstracts Service number. LRI: linear retention index.

## Supplementary Materials

The following are available online at https://www.mdpi.com/2076-3921/9/4/338/s1: Table S1: Composition analysis for fat-filled whole milk powder (FFWMP), skim milk powder (SMP), and infant milk formula (IMF). Each result is the average of 2 replicates.; Table S2: Results of one-way ANOVA followed by the post hoc Tukey test for the colour of the 12 reconstituted milk powders after 4 months in storage. Data are expressed as the mean ± standard deviation (*n* = 3). ^a–k^ Values within a column with different superscripts are statistically different at *p* < 0.001.; Table S3: Fatty acid (FA) composition (g/100 g of FA ± SD; *n* = 3) of infant milk formula (IMF) powder samples (Brands 1–5).
