# Supplementary material for: Correlating Volatile Lipid Oxidation Compounds with Consumer Sensory Data in Dairy Based Powders during Storage
Source: Antioxidants (Basel). 2020 Apr 20;9(4):338. doi: 10.3390/antiox9040338 (PMC7222397; doi:10.3390/antiox9040338)
Supplement: Supplementary file 1 [file antioxidants-09-00338-s001.pdf]

Table S1: Composition analysis for Fat-filled whole milk powder (FFWMP), skim milk powder (SMP) and infant milk formula (IMF). Each result is the average of 2 replicates.

| **Sample** | **Fat %** | **Protein %** | **Lactose %** | **Total solids %** | **True protein %** | **Casein %** |
|---|---|---|---|---|---|---|
| FFWMP (AM) | 3.6 | 3.4 | 5.1 | 13.2 | 3.2 | 2.3 |
| SMP (AM) | 0.03 | 3.7 | 5.6 | 10 | 3.5 | 2.8 |
| IMF (AM) | 2.8 | 1.7 | 7.5 | 12.8 | 1.5 | 1.1 |
| FFWMP (CON) | 3.7 | 3.4 | 5.2 | 12.8 | 3.2 | 2.4 |
| SMP (CON) | 0.03 | 3.8 | 5.6 | 10.2 | 3.6 | 2.9 |
| IMF (CON) | 2.8 | 1.7 | 7.9 | 12.8 | 1.5 | 1.1 |
| FFWMP (ACC) | 3.8 | 3.5 | 5.3 | 13.2 | 3.3 | 2.4 |
| SMP (ACC) | 0.02 | 3.7 | 5.6 | 10 | 3.6 | 2.8 |
| IMF (ACC) | 2.9 | 1.7 | 7.8 | 12.8 | 1.5 | 1.1 |
| FFWMP (HUM) | 3.6 | 3.4 | 5.1 | 12.6 | 3.2 | 2.3 |
| SMP (HUM) | 0.03 | 3.8 | 5.7 | 10.2 | 3.6 | 2.9 |
| IMF (HUM) | 2.8 | 1.6 | 7.8 | 12.7 | 1.5 | 1.1 |

Table S2: Results of One-way ANOVA followed by post hoc Tukey test for the colour of the 12 reconstituted milk powders after 4 months in storage. Data are expressed as mean ± standard deviation ($n$ = 3). Different superscripts within a column indicate significant differences ($p$ = 0.001).

| Sample | L | a (-) | b | *P-value* |
|---|---|---|---|---|
| FFWMP (AM) | 87.62 ± 0.01a | 2.89 ± 0.49a | 6.31 ± 0.07a | *** |
| SMP (AM) | 77.09 ± 0.11b | 5.72 ± 0.44b | 2.48 ± 0.68b | *** |
| IMF (AM) | 84.88 ± 0.02c | 3.10 ± 0.92c | 4.51 ± 0.10c | *** |
| FFWMP (CON) | 86.03 ± 0.01d | 2.68 ± 0.63d | 6.05 ± 0.08d | *** |
| SMP (CON) | 77.52 ± 0.01e | 5.77 ± 0.37b | 2.44 ± 0.33e | *** |
| IMF (CON) | 83.43 ± 0.12f | 3.08 ± 1.07c | 4.20 ± 0.30f | *** |
| FFWMP (ACC) | 87.30 ± 0.01g | 2.89 ± 0.43a | 6.63 ± 0.00g | *** |
| SMP (ACC) | 77.64 ± 0.03e | 5.83 ± 0.08b | 2.65 ± 0.31h | *** |
| IMF (ACC) | 84.89 ± 0.01c | 3.07 ± 0.31c | 4.75 ± 0.31i | *** |
| FFWMP (HUM) | 90.30 ± 0.01h | 2.79 ± 0.34i | 6.26 ± 0.08j | *** |
| SMP (HUM) | 77.46 ± 0.03e | 5.71 ± 0.44b | 2.62 ± 0.48h | *** |
| IMF (HUM) | 84.42 ± 0.01c | 3.14 ± 1.05c | 4.15 ± 0.34k | *** |

Table S3: Fatty acid (FA) composition (g/100 g of FA ± SD; $n$ = 3) of infant milk formula (IMF) powder samples (brand 1–5).

| **Fatty Acids** | **IMF Brand 1** | **IMF Brand 2** | **IMF Brand 3** | **IMF Brand 4** | **IMF Brand 5** | ***P* - Value** |
|---|---|---|---|---|---|---|
| Butyric acid C4:0 | 0.82 ± 0.08 | 0.07 ± 0.01 | 0.06 ± 0.01 | 0.05 ± 0.01 | 0.04 ± 0.01 | <0.001 |
| Caproic acid C6:0 | 0.32 ± 0.01 | 0.20 ± 0.01 | 0.26 ± 0.02 | 0.24 ± 0.02 | 0.02 ± 0.01 | <0.001 |
| Octanoic acidC8:0 | 0.26 ± 0.01 | 1.74 ± 0.07 | 2.36 ± 0.13 | 2.18 ± 0.19 | 0.03 ± 0.01 | 0.03 |
| Decanoic acid C10:0 | 0.44 ± 0.01 | 1.33 ± 0.01 | 1.75 ± 0.01 | 1.63 ± 0.13 | 0.04 ± 0.01 | <0.001 |
| Lauric Acid C12:0 | 1.35 ± 0.01 | 9.51 ± 0.66 | 12.38 ± 0.26 | 11.66 ± 0.89 | 0.21 ± 0.01 | <0.001 |
| Tridecanoic acid C13:0 | 0.01 ± 0.01 | 0.01 ± 0.01 | 0.01 ± 0.01 | 0.01 ± 0.01 | ND | <0.001 |
| Myristic acid C14:0 | 1.83 ± 0.01 | 3.93 ± 0.20 | 4.78 ± 0.05 | 4.58 ± 0.30 | 0.70 ± 0.07 | <0.001 |
| Myristoleic acid C14:1 c9 | 0.15 ± 0.01 | 0.02 ± 0.00 | 0.01 ± 0.01 | 0.01 ± 0.01 | ND | <0.001 |
| Pentadecanoic acid C15:0 | 0.25 ± 0.01 | 0.05 ± 0.00 | 0.05 ± 0.01 | 0.05 ± 0.01 | 0.05 ± 0.01 | <0.001 |
| Palmitic acid C16:0 | 18.97 ± 0.38 | 17.28 ± 0.17 | 14.92 ± 0.09 | 14.77 ± 0.58 | 24.36 ± 1.72 | <0.001 |
| Palmitoleic acid C16:1 c9 | 0.26 ± 0.01 | 0.16 ± 0.01 | 0.14 ± 0.01 | 0.16 ± 0.01 | 0.14 ± 0.01 | <0.001 |
| Heptadecanoic acid C17:0 | 0.14 ± 0.01 | 0.09 ± 0.01 | 0.06 ± 0.01 | 0.07 ± 0.01 | 0.08 ± 0.01 | 0.001 |

| | | | | | | |
|---|---|---|---|---|---|---|
| Stearic acid C18:0 | 4.15 ± 0.23 | 2.56 ± 0.10 | 2.19 ± 0.05 | 2.20 ± 0.02 | 3.30 ± 0.11 | <0.001 |
| Oleic acid C18:1 n9c | 27.54 ± 0.75 | 28.02 ± 0.80 | 27.61 ± 0.47 | 27.54 ± 0.52 | 30.36 ± 1.52 | 0.092 |
| Elaidic acid C18:1 n9t | 3.18 ± 0.18 | 1.59 ± 0.01 | 1.48 ± 0.01 | 1.58 ± 0.21 | 2.23 ± 0.42 | 0.003 |
| Linoleic acid C18:2 n6c | 17.54 ± 0.56 | 13.12 ± 0.32 | 9.97 ± 0.15 | 10.18 ± 0.17 | 15.45 ± 0.82 | <0.001 |
| trans-9,12-octadecadienoate C18:2 n6t | 18.47 ± 1.74 | 16.87 ± 0.22 | 17.91 ± 0.13 | 18.97 ± 2.53 | 19.78 ± 3.78 | 0.736 |
| $\alpha$-Linolenic acid C18:3 n3 | 1.94 ± 0.06 | 1.49 ± 0.05 | 1.74 ± 0.01 | 1.83 ± 0.04 | 1.52 ± 0.07 | 0.001 |
| Gamma Linolenic Acid c18:3 n6 | 0.02 ± 0.01 | 0.10 ± 0.01 | 0.17 ± 0.03 | 0.16 ± 0.04 | 0.08 ± 0.02 | 0.009 |
| Eicosanoic acid C20:0 | 0.24 ± 0.01 | 0.22 ± 0.02 | 0.21 ± 0.01 | 0.21 ± 0.01 | 0.27 ± 0.01 | 0.011 |
| cis-11-Eicosenoic acid C20:1 | 0.24 ± 0.01 | 0.26 ± 0.02 | 0.27 ± 0.01 | 0.28 ± 0.01 | ND | <0.001 |
| Eicosenoic acid C20:2 | 0.03 ± 0.01 | 0.01 ± 0.01 | ND | ND | ND | 0.044 |
| Nervonic acid C24:1 n9 | 0.26 ± 0.02 | 0.21 ± 0.09 | 0.15 ± 0.09 | 0.16 ± 0.01 | 0.20 ± 0.09 | 0.541 |
| Eicosapentaenoic acid C20:5 | 0.05 ± 0.01 | 0.04 ± 0.06 | ND | ND | ND | 0.247 |
| CLA C18:2 c9t11 | 1.55 ± 0.14 | 1.12 ± 0.06 | 1.32 ± 0.01 | 1.48 ± 0.20 | 1.15 ± 0.23 | 0.111 |
